# Experimental model of portal hypertension and esophagogastric varices in minipigs: pressure and endoscopic pilot study

**DOI:** 10.1590/acb370103

**Published:** 2022-03-07

**Authors:** Fauze Maluf-Filho, Alberto Meyer, Pierre Pirchner Mathias Martins, Flávio Henrique Ferreira Galvão, Luiz Augusto Carneiro D’Albuquerque

**Affiliations:** 1 PhD. Endoscopy Unit – Instituto do Câncer do Estado de São Paulo – Department of Gastroenterology, and Laboratório de Transplante e Cirurgia do Fígado – Universidade de São Paulo – Sao Paulo (SP), Brazil.; 2 PhD. Liver and Abdominal Organs Transplantation Division – Department of Gastroenterology, and Laboratório de Transplante e Cirurgia do Fígado – Universidade de São Paulo – Sao Paulo (SP), Brazil.; 3 MD. Department of Gastroenterology – Universidade do Estado do Rio de Janeiro – Rio de Janeiro (RJ), Brazil.; 4 PhD, Full Professor – Liver and Abdominal Organs Transplantation Division – Department of Gastroenterology, and Laboratório de Transplante e Cirurgia do Fígado – Universidade de São Paulo – Sao Paulo (SP), Brazil.

**Keywords:** Hypertension, Portal, Esophageal and Gastric Varices, Portal Vein, Swine, Miniature

## Abstract

**Introduction:**

Portal hypertension still represents an important health problem worldwide. In the search for knowledge regarding this syndrome, experimental studies with animal models have proven to be useful to point the direction to be taken in future randomized clinical trials.

**Purpose:**

To validate the experimental model of portal hypertension and esophagogastric varices in a medium-sized animal.

**Methods:**

This study included five minipigs br1. Midline laparotomy with dissection of the portal vein and production of a calibrated stenosis of this vein was performed. Measurement of pressure in the portal venous and digestive endoscopic were performed before and five weeks after the production of a stenosis.

**Results:**

All animals were 8 months old, average weight of 17 ± 2.5 kg. The mean pressure of the portal vein immediately before the partial ligation of the portal vein was 8.9 + 1.6 mm Hg, with 26.6 + 5.4 mm Hg in the second measurement five weeks later (p < 0.05). No gastroesophageal varices or hypertensive portal gastropathy were seen at endoscopy procedures in our sample at any time in the study.

**Conclusion:**

Portal vein ligation in minipigs has been validated in the production of portal hypertension, but not in the formation of esophageal varices.

## Introduction

Portal hypertension plays a fundamental role in the pathophysiology of complications that lead to decompensation of advanced chronic liver disease, such as ascites, hepatic encephalopathy and hemorrhage due to rupture of gastroesophageal varices. Bleeding from esophageal varices has an accumulated incidence of approximately 10–15% per year, varying according to the degree of liver failure, diameter of the varices and the presence of red signs^1^^-^3. Such event has a 15–25%-mortality rate in the first six weeks after the first episode. If untreated, about 60% of patients who survive the first bleeding episode will rebleed, usually within one or two years^1^^-^3.

Between 1997 and 2002, nearly 4,000 cases of upper gastrointestinal bleeding (UGB) were treated at the Endoscopy Emergency Room of the Hospital das Clínicas, Faculdade de Medicina, Universidade de São Paulo (HCFMUSP), São Paulo, SP, Brazil. Esophageal and gastric varices were the origin of bleeding in 18.6 and 3.6% of these cases, respectively. Together, they share the first place of source UGB, together with peptic ulcer disease^4^. Between 2005 and 2007, 480 patients with UGB caused by rupture of esophageal varices were treated at the same center, with average of 20 cases per month^5^. It is noteworthy that, even with the recommended therapy, mortality caused by esophageal varices rupture in 50 patients seen at HCFMUSP reached 32%^5^^,^6^-^9. This data underlines the importance of continuous refinement of the best therapeutic for the treatment of variceal bleeding.

In the search for knowledge regarding the portal hypertension syndrome, experimental studies with animal models have proven to be essential. The results of these studies are useful to point the direction to be taken in future randomized clinical trials, which are not only difficult to carry out, but longer and more costly. Rodents are the most used animals for experimental models of portal hypertension, probably because their lower cost, simplicity, space requirements and reproducibility^10^. There are several methods capable to reproduce one of the three types of portal hypertension (i.e., prehepatic and posthepatic), each with its own characteristics, results and applications^11^. Biliary tract ligation, hepatic artery embolization with 80% alcohol, infusion of non-radioactive colored tracer microspheres, intoxication by carbon tetrachloride (CCL4) and thioacetamide (TAA) are the most used intrahepatic portal hypertension models, while the gradual occlusion of the inferior vena cava (gOIVC) is the standard model for posthepatic portal hypertension^12^^-^19.

However, when the focus is on the study of esophageal varices, models of prehepatic portal hypertension are the ones of choice. The most used method for this model is the partial portal vein ligation (PPVL). It is a simple, highly reproducible surgery, in which a puncture is performed along the portal vein (19-21G needle), with subsequent partial ligation of this (3-0 silk), leading to the formation of a calibrated stenosis. This is the method of choice for the study of esophageal varices because it produces more pronounced portosystemic collateral circulation in a more prevalent and rapid way (around seven days), compared with other models^10^^,^11^,^20.

When we focus on the study of esophageal varices, only medium-sized animals allow upper digestive endoscopy. A special breed of pig, known as minipig, has been used in numerous experimental studies, due to its characteristics^21^^,^22. This animal model has splanchnic hemodynamics and liver metabolism that are like humans’, in addition to tolerating partial occlusion of the portal vein for up to six months, a period sufficient to analyze the acute and chronic effects of portal hypertension^23^. In addition, they have adequate size, reaching an average weight of 18 kg at 8 months, while other pig breeds reach over 100 kg at the same age. The PPVL method in minipigs was first described by Jensen et al. in 1986. In that study, there was significant increase in portal vein pressure in the five ligated animals, with varices formation after five weeks. The esophagus varices formed exhibited an estimated diameter of 5–7 mm, remaining unchanged at week 24 of the study^24^^-^26.

Our work aimed to validate the experimental model of portal hypertension and esophagogastric varices in a medium-sized animal, the minipig br1 (Minipigs, Pesquisa e Desenvolvimento LTDA), through a calibrated ligation of the portal vein. This will open the field for several other lines of research, in which it is possible to perform diagnostic and therapeutic procedures.

## Methods

This is an experimental study using an animal model, developed in 2014, currently underway at LIM-37 of the HCFMUSP. This study was approved by the Animal Use Ethics Committee (CEUA) of the Research Ethics Committee, Faculdade de Medicina, Universidade de São Paulo, under protocol number 416/13.

### Animal facilities and veterinary care

Five minipigs br1 were included. The animals were provided by Minipigs (Pesquisa e Desenvolvimento LTDA) and transported to the vivarium of the Faculdade de Medicina, Universidade de São Paulo, where they were kept in conventional standard individual pens, at room temperature. The stalls were cleaned and disinfected twice a day, according to the department protocol.

The animals were weighed on arrival and fasted of solid food for 24 hours, receiving water as needed. The next day, they were transported to the LIM-37 using a specific cart and accompanied by a vivarium technician. Before the procedure, the animals were anesthetized.

## Anesthetic procedure

General anesthesia was administered by an anesthesiologist. Initially, the animals were sedated (Table 1), using venoclysis in the ear. After anesthetic induction, the pigs were intubated and the endotracheal tube coupled to an inhalation anesthesia device (Takaoka^®^ brand, model Origami Ergo System), with a universal vaporizer, using isoflurane anesthetic liquid for maintenance of general anesthesia, along with the intravenous anesthetics listed in Table 1. The animals were monitored throughout the procedure, with continuous measurement of oxygen saturation by hemoglobin and heart rate with a Dixtal^®^ monitor (model DX 2010). During anesthesia, venous access was performed in the external jugular for postoperative analgesia.

**Table 1:** Anesthetic medications.

Medication (IV/IM)	Quantity	Quantity for induction (IV)	Quantity (ml) for maintenance (Iv or Inhalation)	Quantity for pre-anesthetics (IM)
Thiopental 2.5%	2 bottles	3 to 4 mg/kg	3 mL every 30 minutesor 6 mL for hours	
Fentanyl 50 mcg/ml	5 mL	2 to 10 mcg/kg	2 mL every 1 hour	
Ketamine 50 mg/mL	1 bottle		1 mL if required	1-2 mg/kg
Xylazine 23.32 mg/mL	2 mL	0.5 to 1.5 mg/kg	1 mL if required	1 mL
Lidocaine 2%	1 mL			1-1.5 mg/kg
Atropine 0.25 mg/mL	1 mL		1 mL if required	
Isoflurane 0.5%	About 30 mL		As indicated by theanesthesia equipment	

IV: intravenous; IM: intramuscular.

### Surgical procedure and pressure measurements of the portal venous system

The abdominal cavity was accessed by midline laparotomy, with subsequent dissection of the portal vein. After dissection, a catheter was introduced into the splenic vein and advanced to the portal vein, allowing the measurement of pressure in the portal venous system. Three successive pressure measurements were taken. We then placed a 3-mm diameter catheter parallel to the portal vein and ligated the two structures with a 3-mm teflon band (ASD-Bard^®^). After removal of the catheter, we produced a calibrated stenosis of the portal vein. The stenosis was proximal to the tip of the catheter previously introduced for pressure measurement. The other end of the catheter was adapted to a port-a-cath^®^ system and implanted subcutaneously in the anterior abdominal wall. The duration of the procedure ranged from 40 to 50 minutes.

Five weeks after the operation, the animals were again anesthetized, following the protocol described before, in order to perform three successive splenic vein pressure measurements upstream of the stenosis through the puncture of the port-a-cath^®^ with a 19G needle.

### Endoscopic procedures

The upper digestive endoscopy exams, for evaluation and photographic recording of esophagogastric varices and portal hypertensive gastropathy, were performed at two different times. The first exam, with the animal already anesthetized, was performed immediately before the surgical approach. The second followed a period of five weeks, right after the second measurement of the pressure of the portal venous system.

### Post-operative care

After returning from the first anesthesia, the animals were transported to the swine maintenance sector of the Bioterium Center, using a specific cart accompanied by a *vivarium* technician. In the first four days after surgery, the animals received tramadol, 4 mg/kg, every 12 hours, through an intravenous catheter in the external jugular vein.

### Euthanasia protocol

Still under general anesthesia, euthanasia was performed by intravenous injection of 10 mL potassium chloride. The animals received the necessary measurements for their disposal according to the Waste Disposal Guidebook in the Hospital das Clínicas, Faculdade de Medicina, Universidade de São Paulo System, which follows Resolution number 306, dated December 7, 2004, of the National Health Surveillance Agency and the Resolution of the National Council Environment, number 358, dated April 29, 2005.

### Statistical analysis

Qualitative variables were described in absolute numbers (n) and frequency (%). Quantitative variables were described as means (± standard deviation). For analysis of quantitative variables dependents, paired t-test was used. Significance level was defined as p < 0.05. Statistical analysis was performed using the IBM Statistical Package for the Social Sciences (SPSS) for Windows software, Version 24.0 (Armonk, NY, United States of America).

## Results

A total of five minipigs br1 was included in our experiment. All animals were 8 months old, with an average weight of 17 ± 2.5 kg. The five animals (100%) were successfully submitted to surgical intervention for partial ligation of the portal vein. The entire cohort survived and returned for a new procedure after five weeks, with subsequent euthanasia.

The mean pressure of the portal vein immediately before the partial ligation of the portal vein was 8.9 ± 1.6 mm Hg, with 26.6 ± 5.4 mm Hg in the second measurement five weeks later (p < 0.05). No gastroesophageal varices or hypertensive portal gastropathy were seen at endoscopy procedures in our sample at any time in the study. The data are presented in Table 2.

**Table 2:** Animal weight, portal vein pressure and endoscopic findings.

Pig	Weight (kg)	PP1	PP2	PP3	MPP	PP1’	PP2’	PP3’	MPP’	Esophageal Varices	Gastric Varices
1	19	8	9	9	8.67	26	27	26	26.3	No	No
2	15	7	7	9	7.67	24	23	23	23.3	No	No
3	14	8	7	8	7.67	25	24	23	24	No	No
4	17	9	9	8	8.67	22	25	23	23.3	No	No
5	20	11	12	12	11.7	35	35	38	36	No	No

PP1: first measurement of portal pressure – before ligation; PP2: 2ª measurement of portal pressure – before ligation; PP3: ٣ª measurement of portal pressure – before ligation; PP1’: 1ª measurement of portal pressure – after ligation; PP2’: 2ª measurement of portal pressure – after ligation; PP3’: 3ª measurement of portal pressure – after ligation; MPP: mean portal pressure – before ligation; MPP’: mean portal pressure – after ligation.

## Discussion

This is the first study that aimed to validate the use of the partial portal vein ligation technique to induce portal hypertension and esophageal varices formation in minipigs. This method was described by Jensen *et al*.^23^ in the 1980s. In our study, we observed that this technique produced significant portal hypertension (26.6 + 5.4 mm Hg p < 0.05) in our sample (five Brazilian br1 minipigs), but without formation of esophageal varices.

The validation and development of this animal model, especially in pigs, are currently important beyond the study of portal hypertension. Partial portal vein ligation, even if selective, has been widely used in cancer surgery, specifically in extensive hepatectomies for the treatment of liver metastases. Its importance lies in the fact that it induces rapid liver hypertrophy contralateral to the ligated segment, thus reducing mortality by acute liver failure^27^^,^28. Therefore, better knowledge of the morbidity of this technique, particularly as it relates to the formation of esophageal varices, is specially significant.

About 15 years after the initial description of the method, the same Danish group evaluated the role of recombinant human epidermal growth in preventing complications secondary to esophageal varices sclerosis^23^. This study included 18 minipigs, all of which underwent partial ligation of the portal vein. After five weeks, this technique replicated the previous results, with the development of portal hypertension and formation of esophageal varices^29^. Similar results were also found in other animal species, ranging in size from small, such as rodents (with esophageal varices identified by portography), to medium, such as dogs^30^^-^34.

However, the formation of esophageal varices was not identified in all species. In a study by Inglés *et al*.^35^ using cats, in seven of the animals, portal stenosis was produced by applying an Ameroid constrictor. This led to portal hypertension in all animals in the fourth week, but without the formation of esophageal varices. Thus, the capacity of this technique to produce the formation of significant portal hypertension in different animal species is indisputable. The same conclusion is not valid for the formation of esophageal varices.

The main hypothesis for similar levels of portal hypertension causing or not esophageal varices in different animal models is related to the anatomical differences of the portal system among species. However, this does not apply to our work, since human and porcine portal system anatomies are similar, even when analyzing the morphological and microstructural characteristics of the portal vein^35^^-^38. We believe that other factors, such as the differences in the physiology and hemodynamics of the portal system among animal species, also influence the formation of esophageal varices. An example would be the predilection for different routes of portosystemic shunt. The most prevalent and prominent in humans is the coronary vein shunt, which is directly related to the formation of esophageal varices, as it drains the submucosal and paraoesophageal veins. However, the same may not hold for some animal models, which would account for an absence or lower prevalence of esophageal varices formation^39^.

Another factor that may play a relevant role in the formation of esophageal varices is the thickness of the esophageal mucosa and submucosa. Therefore, species that have such thicker esophageal layers would tend to produce less engorgement of varicose collateral veins. Ultimately, the minipigs used in Jensen *et al*.^25^ research were the Gottingen minipigs. There are at least 14 pure breeds of minipigs, and Gottingen is one of them. We speculate that the differences between minipig br1 and the Gottingen breed may have also played a role in our findings, even though there are no studies comparing the portal vascular anatomy between these two breeds. 

An issue that also deserves reflection when we analyze the absence of esophageal varices formation in our study, compared to previous studies by Jensen *et al*.^25^, is the subjectivity of the diagnosis of esophageal varices, especially those of small caliber. In two of their studies, there was a description of the formation of small-caliber varices, with a luminal projection of 3 mm^25^^,^29. In this diameter, the diagnosis of esophageal varices via upper digestive endoscopy has great inter- and intra-observer variability. Therefore, it is fair to conjecture that the formation of esophageal varices in this model may not be as prevalent as we initially believed.

This study has a limitation related to the small number of animals included in the experiment and few results. Other limitation is that the data obtained during the study are not supported by macroscopic or endoscopic photos. On the other hand, it has the advantage of being pioneer in the initiative of developing studies with medium-sized animals in our environment.

## Conclusion

Portal vein ligation has been validated in the production of portal hypertension, but not in the formation of esophageal varices in the minipigs br1. The authors have been evaluated the possibility of associating, in a future investigation, splenectomy with the base model (portal vein ligation) in order to increase the portal flow to the periesophageal veins. This would improve the br1 model to obtain a reliable and reproducible esophageal varix model.

## References

[1] Garcia-Tsao G, Abraldes JG, Berzigotti A, Bosch J. (2017). Portal hypertensive bleeding in cirrhosis: risk stratification, diagnosis, and management: 2016 Practice Guidance by the American Association for the study of liver diseases. Hepatology.

[2] Baiges A, Hernández-Gea V, Bosch J. (2018). Pharmacologic prevention of variceal bleeding and rebleeding. Hepatol Int.

[3] Nett A, Binmoeller KF (2019). Endoscopic management of portal hypertension-related bleeding. Gastrointest Endosc Clin N Am.

[4] Marques PO, Maluf-Filho F, Kumar A, Matuguma S, Sakai P, Ishioka S (2008). Long term outcomes of acute gastric variceal bleeding in 48 patients following treatment with cyanoacrylate. Dig Dis Sci..

[5] Luz GO, Maluf-Filho F, Matuguma SE, Hondo FY, Ide E, Melo JM, Cheng S, Sakai P. (2011). Comparison between endoscopic sclerotherapy and band ligation for hemostasis of acute variceal bleeding. World J Gastrointest Endosc.

[6] De Franchis R (2015). Baveno VI Faculty. Expanding consensus in portal hypertension: Report of the Baveno VI Consensus Workshop: stratifying risk and individualizing care for portal hypertension. J Hepatol.

[7] Cabrera L, Tandon P, Abraldes JG. (2017). An update on the management of acute esophageal variceal bleeding. Gastroenterol Hepatol.

[8] Bittencourt PL, Strauss E, Farias AQ, Mattos AA, Lopes EP (2017). Variceal bleeding: update of recommendations from the Brazilian Association of Hepatology. Arq Gastroenterol.

[9] Karstensen JG, Ebigbo A, Bhat P, Dinis-Ribeiro M, Gralnek I, Guy C, Le Moine, Vilmann P, Antonelli G, Ijoma U, Anigbo G, Afiheni M, Duduyemi B, Desalegn H, De Franchis, Ponchon T, Hassan C, Aabakken L. (2020). Endoscopic treatment of variceal upper gastrointestinal bleeding: European Society of Gastrointestinal Endoscopy (ESGE) Cascade Guideline. Endosc Int Open.

[10] Königshofer P, Brusilovskaya K, Schwabl P, Reiberger T. (2019). Animal models of portal hypertension. Biochim Biophys Acta Mol Basis Dis.

[11] Bosch J, Iwakiri Y. (2018). The portal hypertension syndrome: etiology, classification, relevance, and animal models. Hepatol Int.

[12] Yanguas SC, Cogliati B, Willebrords J, Maes M, Colle I, van den Bossche B, de Oliveira CPMS, Andraus W, Alves VAF, Leclercq I, Vinken M (2016). Experimental models of liver fibrosis. Arch Toxicol.

[13] Klein S, Schierwagen R, Uschner FE, Trebicka J. (2017). Mouse and rat models of induction of hepatic fibrosis and assessment of portal hypertension. Methods Mol Biol.

[14] Avritscher R, Wright KC, Javadi S, Uthamanthil R, Gupta S, Gagea M, Bassett RL, Murthy R, Wallace MJ, Madoff DC (2011). Development of a large animal model of cirrhosis and portal hypertension using hepatic transarterial embolization: a study in swine. J Vasc Interv Radiol.

[15] Wang L, He FL, Liu FQ, Yue ZD, Zhao HW. (2015). Establishment of a hepatic cirrhosis and portal hypertension model by hepatic arterial perfusion with 80% alcohol. World J Gastroenterol.

[16] Jin W, Deng L, Zhang Q, Lin D, Zhu J, Chen Y, Chen B, Li J. (2010). A canine portal hypertension model induced by intra-portal administration of Sephadex microsphere. J Gastroenterol Hepatol.

[17] Scholten D, Trebicka J, Liedtke C, Weiskirchen R. (2015). The carbon tetrachloride model in mice. Lab Anim.

[18] Staňková P, Kučera O, Lotková H, Roušar T, Endlicher R, Cervinková Z (2010). The toxic effect of thioacetamide on rat liver in vitro. Toxicol In Vitro.

[19] Shen B, Zhang Q, Wang X, Xu H, Zu M, Wu M, Gao Z, Wang W, Xiao J, Wang Y. (2014). Development of a canine model with diffuse hepatic vein obstruction (Budd Chiari syndrome) via endovascular occlusion. Mol Med Rep.

[20] Rodrigues DA, da Silva, Serigiolle LC, Fidalgo Rde, Favero SS, Leme PL. (2015). Constriction rate variation produced by partial ligation of the portal vein at pre-hepatic portal hypertension induced in rats. Arq Bras Cir Dig.

[21] Mariano M. (2003). Miniature swine (minipig) in biomedical experimental research: the Minipig br1. Acta Cir Bras.

[22] Swindle MM. (2007). Swine in the laboratory: surgery, anesthesia, imaging and experimental techniques.

[23] Jensen LS, Krarup N, Larsen JA, Juhl C, Nielsen TH. (1986). Effect of acute portal hypertension on hepatosplanchnic hemodynamics and liver function. Scand J Gastroenterol.

[24] Jensen LS, Krarup N, Larsen JA, Juhl C, Nielsen TH, Dybdahl H. (1987). Effect of endoscopic sclerotherapy of esophageal varices on liver blood flow and liver function. An experimental study. Scand J Gastroenterol.

[25] Jensen LS, Krarup N, Juhl CO, Nielsen TH, Larsen JA (1989). Endoscopic, portographic, and hemodynamic evaluation of prolonged propranolol administration in pigs with experimental portal hypertension and esophageal varices. Scand J Gastroenterol.

[26] Jensen LS, Krarup N, Larsen JA, Juhl C, Nielsen TH, Dybdahl H. (1987). Chronic portal venous hypertension. The effect on liver blood flow and liver function and the development of esophageal varices. Scand J Gastroenterol.

[27] ALPPS (2020). (ALPPS) registry: what have we learned?. Gut Liver.

[28] Zhang GQ, Zhang ZW, Lau WY, Chen XP (2014). Associating liver partition and portal vein ligation for staged hepatectomy (ALPPS): a new strategy to increase resectability in liver surgery. Int J Surg.

[29] Juhl CO, Vinter-Jensen L, Jensen LS, Nexø E, Djurhuus JC, Dajani EZ (1994). Systemic treatment with recombinant human epidermal growth factor accelerates healing of sclerotherapy-induced esophageal ulcers and prevents esophageal stricture formations in pigs. Dig Dis Sci.

[30] Nishida R, Inoue R, Takimoto Y, Hamashima H, Kita T (1995). Endoscopic sclerotherapy in a rat model of esophageal varices. Scand J Gastroenterol.

[31] Tanoue K, Kitano S, Hashizume M, Wada H, Sugimachi K (1991). A Rat model of esophageal varices. Hepatology.

[32] Jensen DM, Machicado GA, Tapia JI, Kauffman G, Franco P, Beilin D (1983). A reproducible canine model of esophageal varices. Gastroenterology.

[33] Chen Y, Zhang Q, Liao Y, Guo F, Zhang Y, Zeng Q, Jin W, Shi H, Zhou M. (2009). A modified canine model of portal hypertension with hypersplenism. Scand J Gastroenterol.

[34] Lin D, Wu X, Ji X, Zhang Q, Lin Y, Chen W, Jin W, Deng L, Chen Y, Chen B, Li J (2012). A novel canine model of portal vein stenosis plus thioacetamide administration-induced cirrhotic portal hypertension with hypersplenism. Cell Biochem Biophys.

[35] Inglés AC, Légaré DJ, Lautt WW. (1993). Development of portacaval shunts in portal-stenotic cats. Can J Physiol Pharmacol.

[36] Nykonenko A, Vávra P, Zonča P. (2017). Anatomic peculiarities of pig and human liver. Exp Clin Transplant.

[37] Zhang Y, Huang T, Wang P, Li W, Yu M. (2005). Comparison of morphology and microstructural components of hepatic portal vein between human and pig. J Huazhong Univ Sci Technolog Med Sci.

[38] He XJ, Huang TZ, Wang PJ, Peng XC, Li WC, Wang J, Tang J, Feng N, Yu MH. (2012). Morphological and biomechanical remodeling of the hepatic portal vein in a swine model of portal hypertension. Ann Vasc Surg.

[39] Leite AF, Mota A, Chagas-Neto FA, Teixeira SR, Elias J, Muglia VF (2016). Acquired portosystemic collaterals: anatomy and imaging. Radiol Bras.

